# Methodology challenges in studying human gut microbiota – effects of collection, storage, DNA extraction and next generation sequencing technologies

**DOI:** 10.1038/s41598-018-23296-4

**Published:** 2018-03-23

**Authors:** Marina Panek, Hana Čipčić Paljetak, Anja Barešić, Mihaela Perić, Mario Matijašić, Ivana Lojkić, Darija Vranešić Bender, Željko Krznarić, Donatella Verbanac

**Affiliations:** 1 University of Zagreb School of Medicine, Center for Translational and Clinical Research, Šalata 2, 10000 Zagreb, Croatia; 2 MRC London Institute of Medical Sciences, Du Cane Road, London, W12 0NN UK; 3 Croatian Veterinary Institute, Department for Virology, Savska cesta 143, 10000 Zagreb, Croatia; 4 University Hospital Zagreb, Department of Internal Medicine, Unit of Clinical Nutrition, Kišpatićeva 12, 10000 Zagreb, Croatia; 5 University of Zagreb School of Medicine, Department of Internal Medicine, Kišpatićeva 12, 10000 Zagreb, Croatia

## Abstract

The information on microbiota composition in the human gastrointestinal tract predominantly originates from the analyses of human faeces by application of next generation sequencing (NGS). However, the detected composition of the faecal bacterial community can be affected by various factors including experimental design and procedures. This study evaluated the performance of different protocols for collection and storage of faecal samples (native and OMNIgene.GUT system) and bacterial DNA extraction (MP Biomedicals, QIAGEN and MO BIO kits), using two NGS platforms for 16S rRNA gene sequencing (Ilumina MiSeq and Ion Torrent PGM). OMNIgene.GUT proved as a reliable and convenient system for collection and storage of faecal samples although favouring *Sutterella* genus. MP provided superior DNA yield and quality, MO BIO depleted Gram positive organisms while using QIAGEN with OMNIgene.GUT resulted in greatest variability compared to other two kits. MiSeq and IT platforms in their supplier recommended setups provided comparable reproducibility of donor faecal microbiota. The differences included higher diversity observed with MiSeq and increased capacity of MiSeq to detect *Akkermansia muciniphila*, [*Odoribacteraceae*], *Erysipelotrichaceae* and *Ruminococcaceae* (primarily *Faecalibacterium prausnitzii*). The results of our study could assist the investigators using NGS technologies to make informed decisions on appropriate tools for their experimental pipelines.

## Introduction

The scientific interest and mounting knowledge on human gut microbiota have led to many important findings associating the composition of bacterial taxa in the human gastrointestinal tract with disorders from the neurologic, psychiatric, respiratory, cardiovascular, gastrointestinal, hepatic, autoimmune, metabolic and oncologic spectra^1,2^. The main source of the information regarding human gut microbiota originated from the analyses of the faeces. The importance of this easily available metabolic waste comes from its microbial content found to hold the potential in diagnosis, disease prediction and therapeutic intervention. This justifies it being considered a “virtual organ”^3^ or a human tissue^4,5^. The analyses of human intestinal microorganisms were, until recently, performed by culture-dependent methodologies, limiting the outputs to the cultivable species. The availability of novel tools, primarily next-generation sequencing (NGS), enabled the assessment of genes and genomes contained within complex microbial communities. The most widely used NGS method for the taxonomic and phylogenetic evaluation of bacterial community composition relies on 16S rRNA gene amplicon analysis^4,6–9^. However, detected composition of the faecal bacterial community can be affected by experimental design and procedures, including sampling and storage protocol as well as DNA extraction method.

Sample collection procedures represent one of the first crucial steps that ensure integrity and stability of the collected material. Although an  early study claimed no difference in community structure upon collection under field conditions^10^, subsequent reports showed importance of the collection procedure^11–14^ with accuracy increased by prompt sample downstream processing (within 2–3 hours^11–14^), immediate stabilization^12,13,15^ and appropriate storage conditions^11–14,16,17^. Faeces represents one of the most complex biological materials for bacterial DNA isolation as it also contains remnants of human DNA, food DNA and many inhibitors hampering subsequent PCR amplification and NGS procedures^18^. DNA extraction from faeces represents the essential step in obtaining good quality DNA and ensuring accurate identification of microbial composition and relative abundance^19^. It is therefore critical to evaluate the available methodologies for bacterial DNA extraction from faeces and optimize protocols and procedures that would provide sufficient amount, purity and integrity of the DNA, thus obtaining high quality sample for further studies.

During the last decade sequencing tools have gradually shifted from conventional Sanger sequencing technology to NGS^9,20^ and currently available technologies with sequencers from Pacific Bioscience, Roche, Thermo Fischer Scientific and Illumina, have all been successfully applied for the analyses of complex biological samples^7,9,19,21,22^. NGS is used for determining bacterial composition in a sample based on DNA fragment detection from the total DNA isolate. Although shotgun sequencing provides the most complete information on the entire gene pool within the sample, the high amount of generated data requires substantial bioinformatic efforts in sequence assembly, mapping and analyses. In many studies, both clinical and environmental, sequencing of 16S rRNA gene amplicons, covering variable regions of the gene, is the method of choice for the analyses of bacterial community composition, providing cost-effectiveness, sufficient resolution and sequencing depth^1,7,8,20^. The 16S rRNA gene is approximately 1500 bp in size and consists of nine variable regions separated by conserved regions^19,23^. Illumina and Thermo platforms with their MiSeq and Ion Torrent (IT) Personal Genome Machine (PGM) benchtop sequencers are increasingly being used for 16S rRNA-based analyses of diverse bacterial populations^7,9,19,21,22^. Although both Illumina and IT platforms sequence DNA by detecting the nucleotide addition during DNA synthesis, the two platforms function on different principles, which could affect their performance and ultimately result in variation of the obtained data^7,9,24–27^.

As each step in the experimental pipeline introduces variation influencing the final output, there is an unmet need for standardization of methodology which would enable reliable and reproducible analysis of valuable human biological samples for studying gut microbiota. This study evaluated the performance of different protocols for collection and storage of faeces (native versus OMNIgene.GUT system) as well as bacterial DNA extraction (with MP Biomedicals, Qiagen and MO BIO commercial kits) using the manufacturer recommended methodology for 16S rRNA gene sequencing on two commercially available platforms (Ilumina MiSeq and IT PGM).

## Results

**Figure 1:**
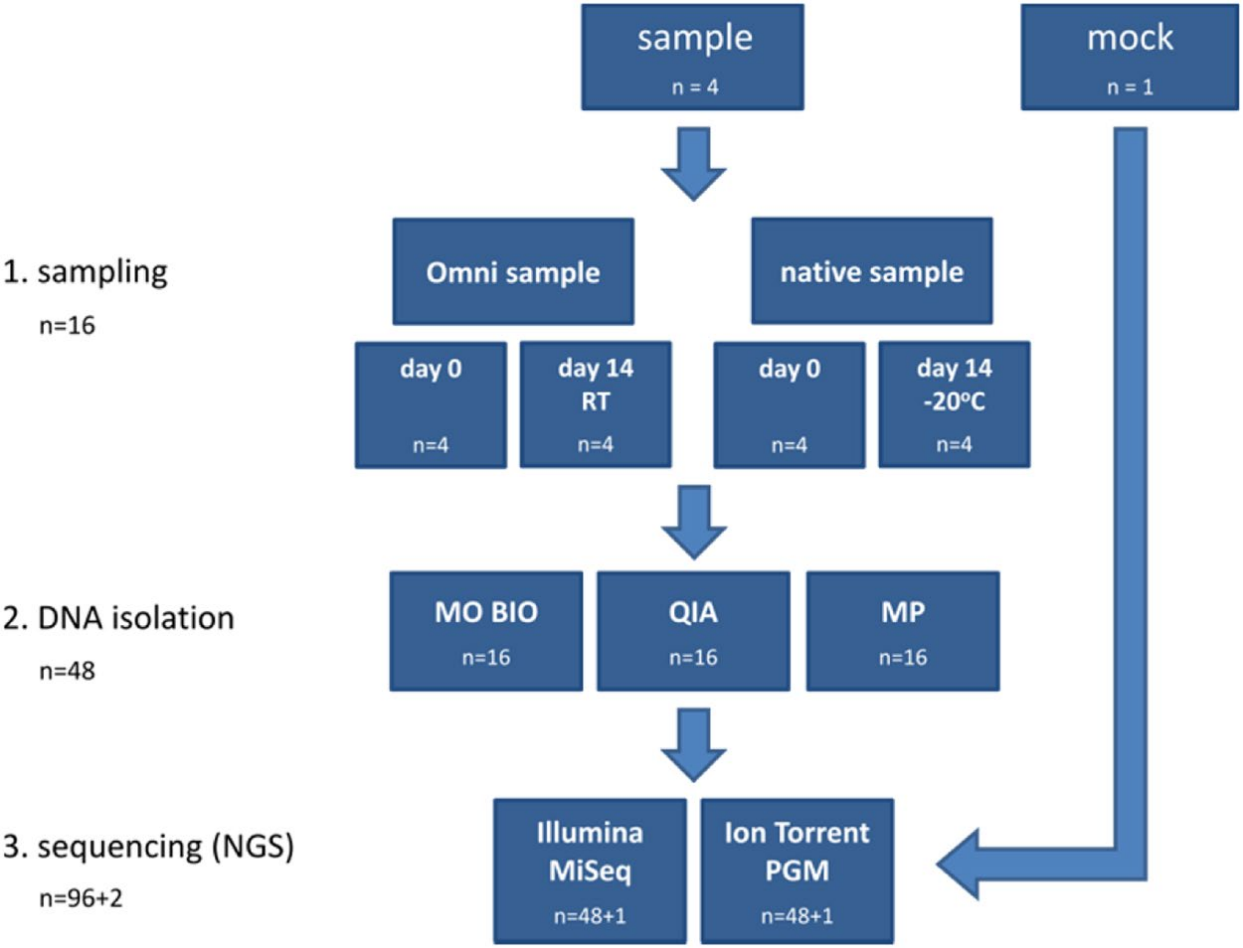
Experimental design. Schematic representation of procedures and conditions for isolation and analysis of microbial DNA from faeces. The figure presents two different sample collection methods, two different storage conditions/sampling time points, three different DNA extraction kits, as well as two different sequencing platforms. Omni = OMNIgene.GUT, RT = room temperature. +1/+2 refer to non-faecal mock samples.

Stool samples from 4 individuals were collected and processed as described in Fig. 1, yielding a total of 96 donor and two mock sample sequences.

### DNA yield and quality

**Figure 2:**
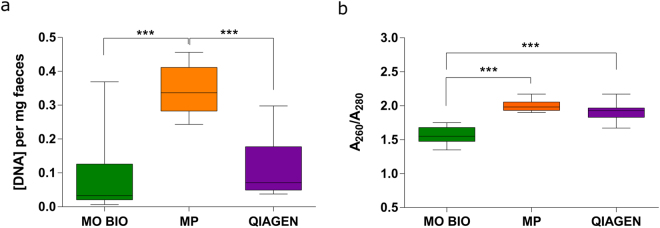
Yield and purity of extracted DNA varies between isolation kits. (**a**) DNA yield and (**b**) quality obtained using different DNA extraction kits (n = 16 samples per kit). DNA yield is expressed as DNA concentration (ng/μl) normalized by quantity of faeces used for extraction. Bonferroni-adjusted unpaired, two-sided t-test: ***p < 0.001.

Summary of DNA isolation methods and obtained DNA concentrations per sampling parameter are given in Supplementary Table 2. The yield and quality of DNA differed between kits, with the average yield and the estimated purity of DNA, both of which varied between kits in the MP > QIA > MO BIO order (Fig. 2). Extracted DNA concentrations were normalized by faecal weight (Fig. 2a). MP yielded approximately three times more DNA than MO BIO and QIA kits (0.34 ± 0,018, 0.09 ± 0.03 and 0.12 ± 0.02 DNA (ng/μl) per mg faeces for MP, MO BIO and QIA, respectively). The best A_260_/A_280_ absorbance ratio was obtained with MP and QIA, with mean values of 2.00 and 1.91, respectively, while mean ratio for MO BIO was significantly lower at 1.55 (Fig. 2b).

A slightly higher DNA yield was obtained when using OMNIgene.GUT collection system compared to the conventional faeces collection at both sampling time points (Supplementary Fig. 1).

### Next generation sequencing – platform comparison

A total of 48 human faecal microbiota DNA samples were obtained, amplified and profiled by 16S RNA amplicon sequencing using Illumina MiSeq (V3-V4 region) and Ion Torrent PGM (V2, V4, V8 and V3, V6-V7, V9 regions). Different primer sets were used to enable comparison of supplier recommended protocols.

**Figure 3:**
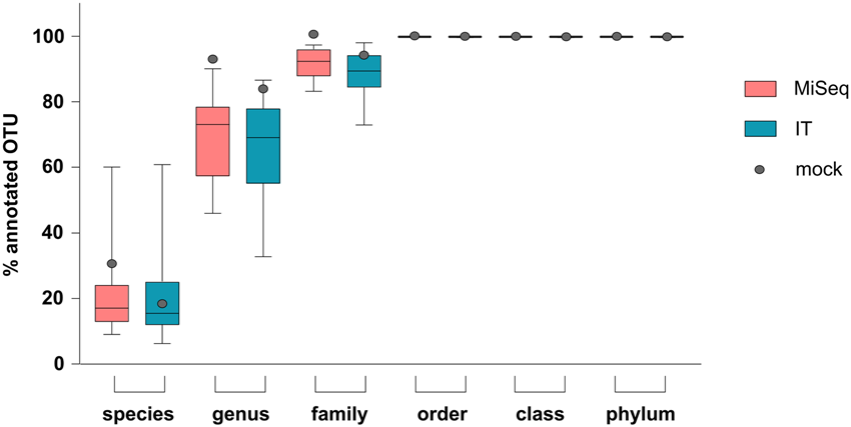
Annotated OTUs per platform at all taxonomic levels. Percentage (%) of annotated OTUs on each taxonomic level for both Illumina MiSeq (MiSeq) and Ion Torrent PGM (IT) sequencing platforms.

Clustering and annotation of OTUs was performed by the same pipeline for both sequencing platforms. Average number of counts per sample was 257.806 ± 23.076 for MiSeq and 110.512 ± 7.529 for IT. A comparison of OTU assignment on each taxonomic level (from species to phylum) revealed that, all OTUs were assigned from phylum to order level for both platforms. At lower taxonomic levels, however, higher proportion of the assigned OTUs was obtained for samples sequenced with MiSeq than those sequenced with IT (Fig. 3).

### Microbiota content complexity

Further insight into the effects introduced by the variations of the experimental pipeline was gained through detailed taxa level analyses, focusing mainly on phylum and family levels since former provides maximal and latter lowest taxonomic level where >85% of annotation was achieved. The annotation at genus level, although lower and variable, was considered sufficient for further analyses but the species level was not further considered due to its inferior and varying annotation efficiency (Fig. 3).

The first platforms comparison was performed on the mock community sample with even DNA quantities from a defined bacterial community. The level of annotated OTUs for the mock community was comparable to that achieved in faeces samples (Fig. 3). The richness of detected taxa on both platforms was in good agreement with the theoretical distribution of all taxa present. However, the evenness diverged to some extent from the theoretically expected values (Supplementary Fig. 2). At genus level all 17 genera were properly assigned (annotation 92% and 83% for MiSeq and IT, respectively) (Fig. 3). This was not the case for species level where only 7/20 were identified (annotation 30% for MiSeq and 17% for IT).

**Figure 4:**
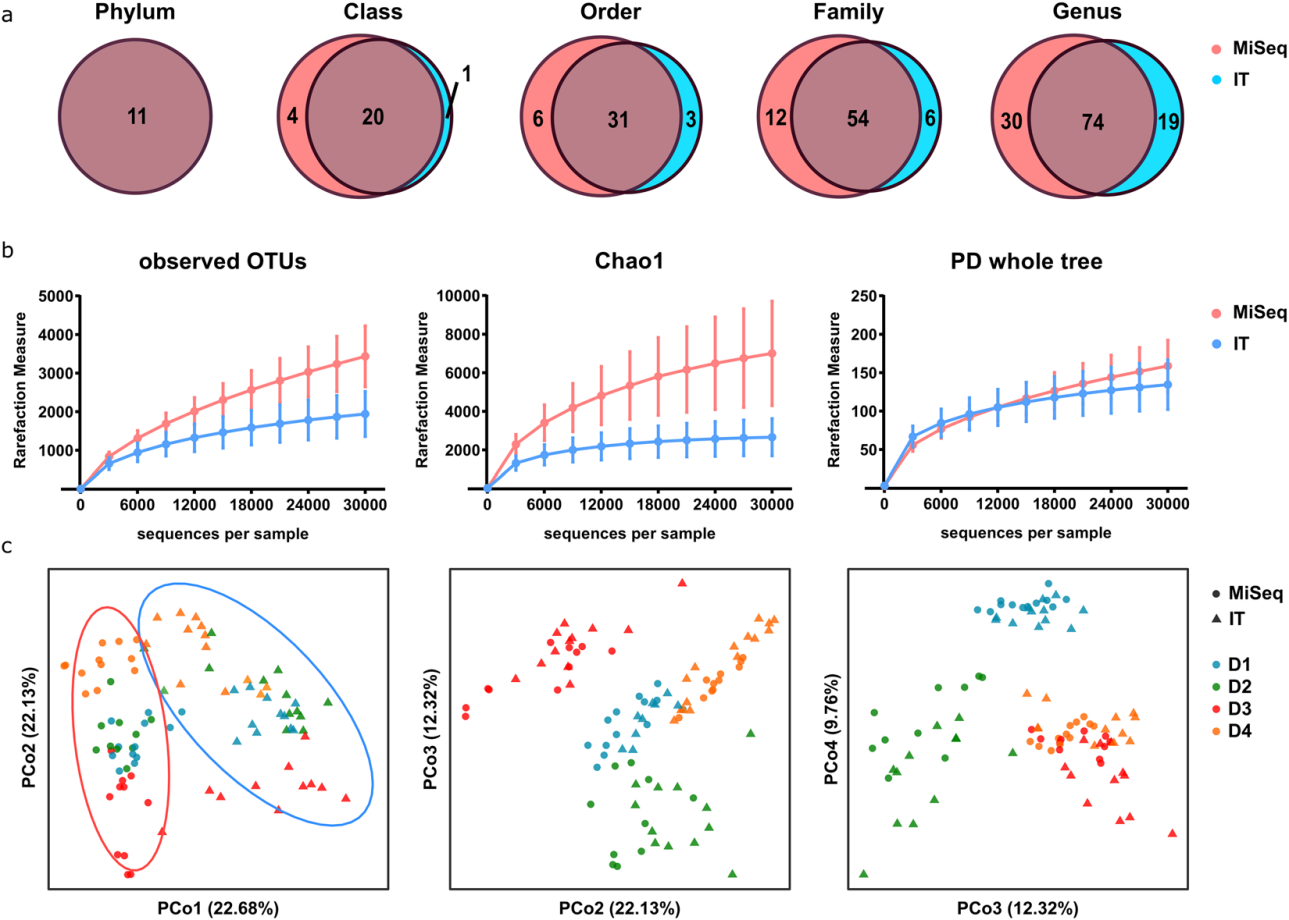
Comparison of diversity between MiSeq and IT platforms. (**a**) Venn diagrams of common and unique number of taxa for individual platform at each taxonomic level, (**b**) alpha diversity indices after rarefaction: observed OTUs, Chao1 index and PD_whole_tree index, (**c**) PCoA plots of beta diversity for first four coordinates marked per platform (MiSeq circled in magenta, IT in turquoise) and per donor.

Next, donor samples were evaluated with respect to total number of commonly annotated taxa at different phylogenetic levels and their diversities (Fig. 4). Raw OTU counts analysis identified many commonly detected, but also a number of platform-specific taxa at each level (Fig. 4a). Diversity indices were calculated to describe the complexity of samples (alpha) and distinguish differences between samples (beta). Alpha diversity was assessed through three indices (Fig. 4b). The more rapid increase in observed OTUs and higher Chao1 (a measure highly favouring singletons and doubletons) with MiSeq than IT was noted. However the difference is lost when phylogenetic distance of detected taxa is taken into account and expressed using PD_whole_tree measure. Additionally, the same indices point to higher diversity in samples where MP was used for DNA extraction (Supplementary Fig. 3). Principle coordinate analysis of beta diversity expressed as weighted UniFrac (Fig. 4c), revealed a major contribution to the differences in diversity first by platform (PCo1) followed by donors (PCo2–4).

### Analysis of microbiota composition

Next, relative abundance of the bacterial phyla detected within the entire sample pool was compared, aggregating all the sequencing data according to platform, thus excluding variations due to other experimental parameters (Supplementary Fig. 4). *Bacteroidetes* and *Firmicutes* phyla accounted for most of the taxon-assigned OTUs identified in all samples (≥90% of all OTUs) (Supplementary Fig. 4a). At the family level, the most abundant taxa identified were *Bacteroidaceae*, *Lachnospiraceae*, *Ruminococcaceae* and *Prevotellaceae* (accounting for >80% of assigned OTUs), displaying comparable abundance ratios between platforms (Supplementary Fig. 4b).

The relative abundance patterns among sample collection procedures and DNA extraction kits were compared on family level (Supplementary Fig. 5). Although OMNIgene.GUT system notably favoured the isolation of *Prevotellaceae* in all tested samples, a good agreement in terms of relative abundance of detected families was found between native and OMNIgene.GUT samples (Omni) (Supplementary Fig. 5a). Variation in *Bacteoridaceae*/*Lachnospiraceae* abundance ratio was observed for samples extracted with different DNA extraction kits (Supplementary Fig. 5b).

**Figure 5:**
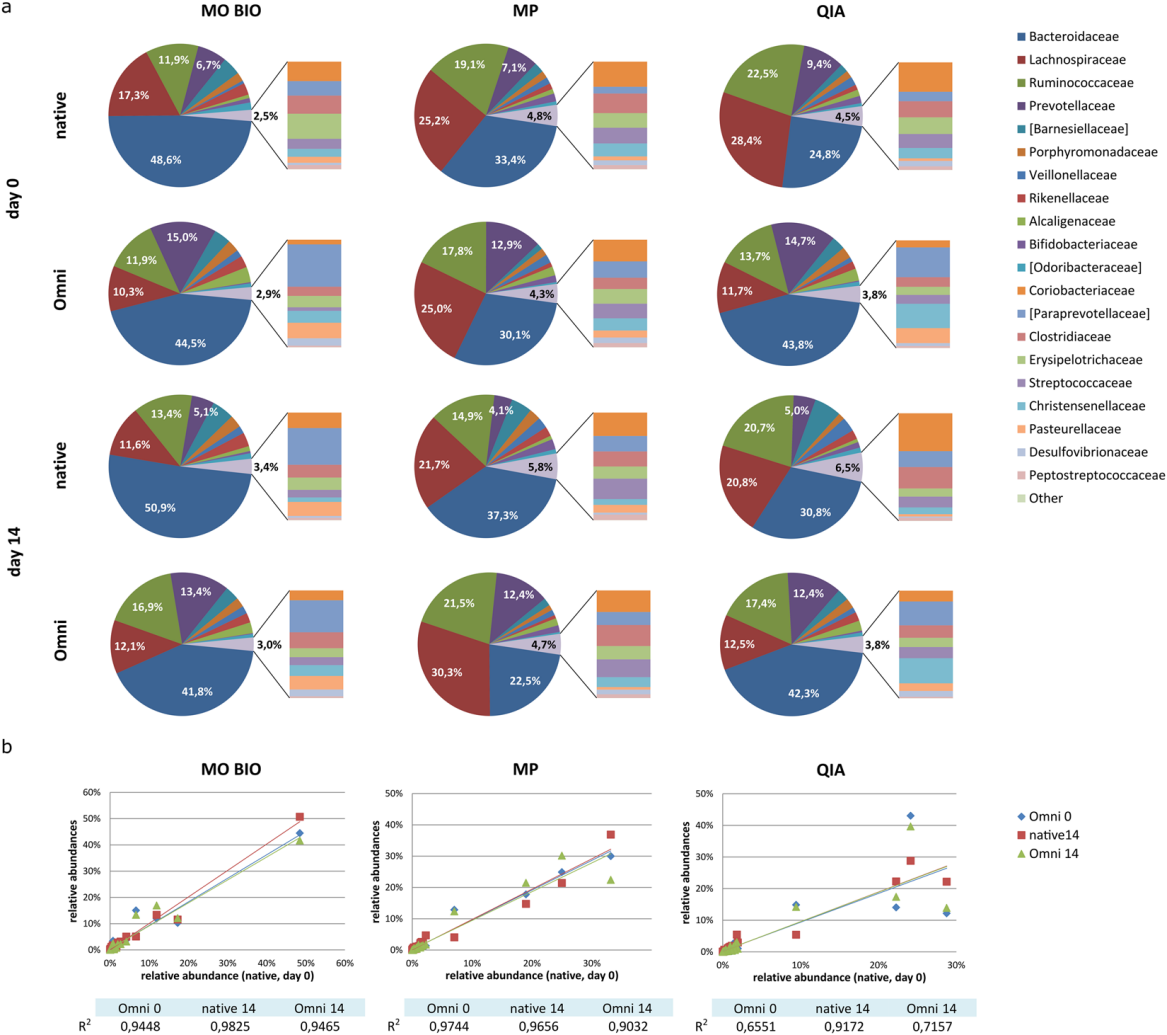
Relative abundance of families depends on sample collection/storage and DNA extraction procedure. Analysis of (**a**) DNA extraction procedures (MO BIO, MP, QIA) with respect to sampling time point (day 0, day 14) and sample collection procedure (native, Omni). Twenty most abundant families are presented, the remainder have been summed as “Other”. 10 major families are displayed on a pie chart, while the remaining ones are shown on a side bar. (**b**) Native 0 sample relative family abundances plotted against corresponding native 14, as well as Omni 0 and Omni 14 relative family abundances for each kit. R^2^ values for linear regression on each time point are displayed in the table below.

Further analysis on phylum (Supplementary Fig. 6) and family (Fig. 5) level included all combinations of experimental parameters tested. Setting the fresh native sample (native 0) as a reference point and comparing the kits (Fig. 5a), it was observed that MO BIO kit tends to provide higher proportion of *Bacteroidaceae* (48.6%) and less *Lachnospiraceae* (17.3%), compared to MP (33.4% and 25.2%) and QIA extraction kit (24.8% and 28.4%, respectively). Comparing the frozen native samples (native 14) with their corresponding reference points revealed the increase in *Bacteoridaceae*/*Lachnospiraceae* ratio and identification of slightly less *Prevotellaceae* across all extraction kits. Collecting the samples with OMNIgene.GUT stabilizing system (Omni 0, Omni 14) tended to increase the *Bacteoridaceae*/*Lachnospiraceae* ratio for QIA and MO BIO extracted samples. Altogether, application of MP and MO BIO resulted in fewer discrepancies across all tested parameters (Fig. 5b). QIA displayed variations between native and Omni samples most notably in the ratio of two dominant families, *Bacteroidaceae* and *Lachnospiraceae*.

**Figure 6:**
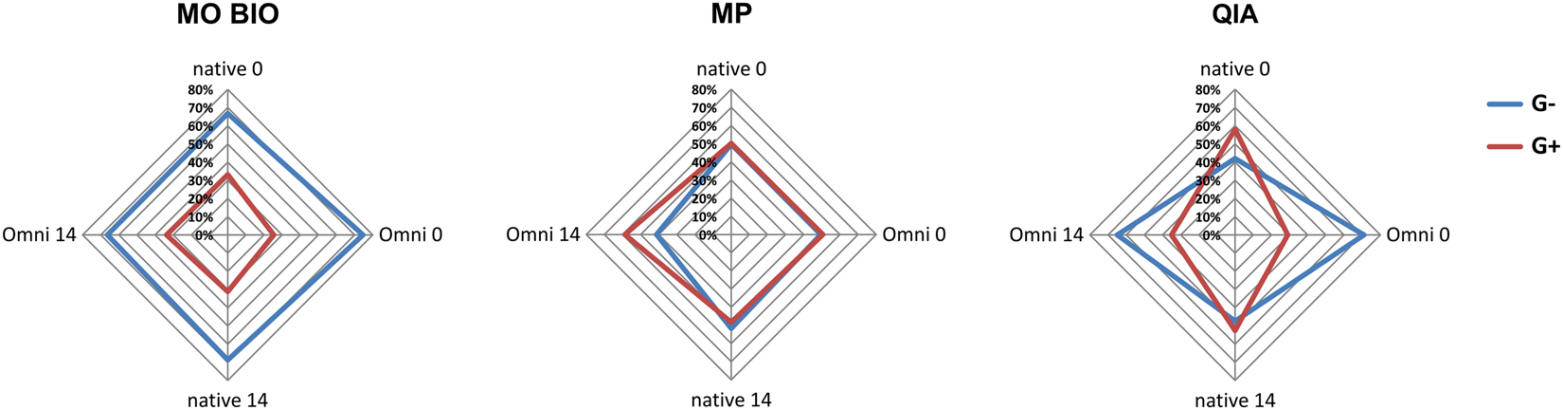
The influence of sample collection/storage protocol and DNA extraction kits on the detected level of Gram+ and Gram− bacteria. The graphs show DNA extraction procedures (MO BIO, MP, QIA) in different sample collection (native, Omni) and storage conditions (day 0, day 14).

Additionally, evaluation of Gram+ and Gram− bacteria extraction efficiency revealed high dependence on collection and storage procedure when using QIA (Fig. 6) as it isolated more G- than G+ bacteria compared to native samples after OMNIGene.GUT stabilisation. MO BIO was less efficient in extracting G+ organisms, while MP kit provided more G+ and an even G+/G− ratio. Unlike QIA, both MO BIO and MP displayed balanced extraction patterns across collection and stabilization procedures.

**Figure 7:**
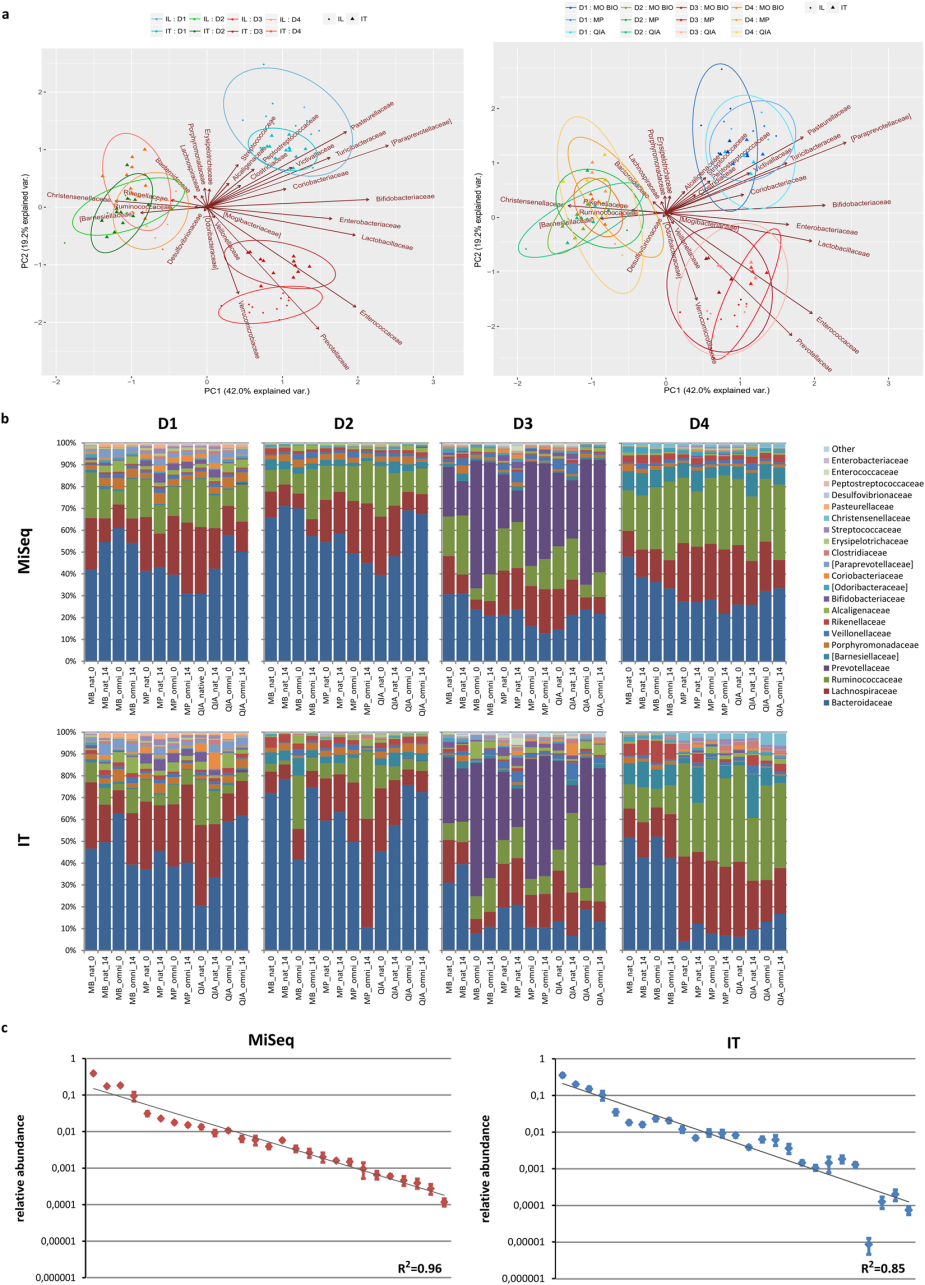
Donor specificity is maintained independent of experimental pipeline. (**a**) PCA bi-plots of clr transformed data on Illumina MiSeq and Ion Torrent PGM sequencing platforms. (**b**) Relative abundance profiles of individual subjects on each platform for top 20 families. (**c**) Relative abundances per platform represented as mean ± SD, with R^2^ values displayed from linear model fitted to log10 values.

The relative abundances of all samples are presented in Fig. 7b. Although each parameter investigated contributed to sample variability, PCA analyses of transformed data (Fig. 7a) confirmed that the most prominent driver of sample clustering was subject specificity, irrespective of any other experimental or platform-related parameter, with each donor maintaining a unique and distinct faecal microbiota profile. Finally, MiSeq relative abundances follow a log-linear trend more closely than IT, with higher correlation and narrower error margins (Fig. 7c).

### Statistical analyses of covariates

The analysis of all covariates presented in Fig. 1 on (clr) transformed data^28^ was performed on each level down to genus.

#### Overall statistics

Wilcoxon rank test was performed for each covariate pair. Statistically significant differences in transformed taxon counts were observed between platforms and kits on family (MiSeq:IT p = 0.026; MOBIO:MP p = 0.049) and genus levels (MiSeq:IT p < 0.001; MOBIO:MP, MOBIO:QIA, MP:QIA all p < 0.001). Omni 0 was found different from both native 0 and native 14 groups of samples only at the genus level (p = 0.003 and 0.004, respectively).

#### Taxon significance

In order to test the effect of each covariate on taxon composition, two tests were performed on clr-transformed data: Kruskal Wallis rank test testing each pair of covariates, and Wilcoxon rank test on Monte-Carlo simulated data testing a covariate vs. all others, e.g. one kit vs. other two. Table 1 lists the main contributors driving differences between platforms and DNA extraction kits at phylum and family levels. Other taxonomic levels and combinations of covariates with statistical significance are available in Supplementary Information (Supplementary Tables 3 and 4).

**Table 1:** Taxon significance between platforms and kits. Only tests yielding Benjamini-Hochberg adjusted p-values less than 0.05 obtained susing Kruskal Wallis test or Wilcoxon rank test, and the matching effect size are shown. Tests performed on centred log ratio normalized data per OTU. Effect size is calculated as the ratio of median difference between groups and largest median variation within groups, values >±0.5 indicate relevance.

	Kruskal-Wallis				Wilcoxon					
	IL:IT	MOBIO:MP	MOBIO:QIA	MP:QIA	IL:IT	effect	MOBIO:other	effect	MP:other	effect
**phylum**										
*Actinobacteria*		< 0.001			0.04	0.336	0.004	0.602	0.001	0.621
*Firmicutes*	0.039	< 0.001		0.035					0.018	0.426
*Lentisphaerae*	0.009				0.037	−0.297				
*Proteobacteria*					0.011	0.434				
*Tenericutes*	0.044				0.044	0.299				
*Verrucomicrobia*	< 0.001				< 0.001	−0.616				
**family**										
*[Mogibacteriaceae]*		< 0.001		0.023			0.045	0.445	0.002	0.641
*[Odoribacteraceae]*	< 0.001				< 0.001	−0.61				
*Bacteroidaceae*	0.001									
*Bifidobacteriaceae*		0.003							0.016	0.348
*Clostridiaceae*		< 0.001							0.01	0.526
*Coriobacteriaceae*		< 0.001					< 0.001	0.733	0.001	0.666
*Desulfovibrionaceae*	< 0.001				0.015	−0.395				
*Erysipelotrichaceae*	< 0.001				0.002	−0.54				
*Lachnospiraceae*	0.019	< 0.001		0.023					0.001	0.635
*Peptostreptococcaceae*		< 0.001							0.001	0.559
*Porphyromonadaceae*	< 0.001				0.028	−0.424				
*Ruminococcaceae*	< 0.001				0.001	−0.557				
*Streptococcaceae*		< 0.001					< 0.001	0.755	0.001	0.674
*Veillonellaceae*	< 0.001				0.014	−0.413				
*Verrucomicrobiaceae*	< 0.001				< 0.001	−0.769				
*Victivallaceae*	0.001				0.007	−0.451				

Platform comparison revealed significant differences among majority of detected phyla. *Actinobacteria*, *Firmicutes*, *Proteobacteria* and *Tenericutes* were found more abundant on IT while *Lentisphaerae* and *Verrucomicrobia* were found more abundant on MiSeq. These differences were statistically significant but the effect sizes were not substantial to support genuine relevance, except *Verrucomicrobia*, which was both highly significant and had a high effect size (a measure quantifying the magnitude of difference between two groups). Even though *Verrucomicrobia* was not a highly abundant phylum, MiSeq detected 10 fold more *Verrucomicrobia* than IT (clr values). It is worth mentioning that the only contribution to *Verrucomicrobia* phylum stems from *Akkermansia muciniphila* species. At the family level, there were ten families that contributed to platform differences, all more abundant on MiSeq, but only four [*Odoribacteraceae*], *Erysipelotrichaceae*, *Ruminococcaceae* and *Verrucomicrobiaceae* have demonstrated high significance and effect sizes. Covariance biplots for organisms with statistical significance (p < 0.05) on family and genus levels (showing highest platform discrepancy) are available in Supplementary Information (Supplementary Fig. 7).

For the DNA extraction kits comparison, MP tends to extract *Actinobacteria* and *Firmicutes* more efficiently than other two kits, while MOBIO is the least efficient in extracting representatives from *Actinobacteria* phylum, confirmed with both the Kruskal Wallis and the Wilcoxon tests. The majority of significantly differing families (Wilcoxon rank test) between kits were found in MP versus other kits (Table 1), with all families more efficiently extracted with MP (1.9–3.2 fold). Kruskal Wallis test revealed that this difference can be accounted for by disparities between MP and MOBIO kits, with QIA coming out as less specific (Table 1). The families with sufficiently high effect size are [*Mogibacteriaceae*], *Clostridiaceae*, *Coriobacteriaceae*, *Lachnospiraceae*, *Peptostreptococcaceae* and *Streptococcaceae*. When MOBIO is compared to other two kits, only three of the above mentioned taxa (*Mogibacteriaceae*], *Coriobacteriaceae*, and *Streptococcaceae*) show significance which stems from lower abundance (by a factor of 2–3.8) these taxa achieve with MOBIO kit (Supplementary Tables 3 and 4).

For the collection-storage combination, Omni 0 was found to enable better extraction of *Betaproteobacteria* class, *Burkholderiales* order, *Alcalligenaceae* family and *Suterella* genus, which was substantiated by Wilcoxon rank test (Supplementary Tables 3 and 4).

## Discussion

With the recent advances in sequencing technologies cost-effective methodologies for microbial composition determination from complex environmental samples became widely available to broad research community. However, each methodological step comprises of multiple technical variables and potentially introduces variation in the final results. These steps include methods of sample collection, stabilisation and storage; DNA extraction and sequencing technology which play an important role in preserving the bacterial content of the sample. Consequently, thorough selection of experimental design and subsequent interpretation of the results needs to be implemented. All this wealth of advanced technological and methodological options exposes the researchers entering the field to plethora of choices only a subset of which is tailored to their particular needs. In addition, the data analysis of the experimental outputs create further level of complexity related to interpretation and comparison across studies in the same research field^27–30^.

Current collection protocols propose the immediate use of fresh faeces or rapid freezing at −80 or −20 °C as optimal^11,12,16,31^. Our results suggest that freezing at −20 °C preserves the specific microbial community composition in faecal samples (Fig. 6 and Supplementary Fig. 5), confirming these literature data. Although it was reported that freezing significantly increased *Firmicutes* to *Bacteroidetes* relative abundance ratio using quantitative PCR analysis^32^, our study identified a decrease in the *Firmicutes*/*Bacteroidetes* ratio, as well as a decrease in the ratio of *Lachnospiraceae* to *Bacteroidaceae*, two major families within *Firmicutes* and *Bacteroidetes* phyla. Also, we found a decline in *Proteobacteria* phylum in frozen faecal samples. These changes, however, were not found statistically relevant. The use of preservative buffers and nucleic acid stabilizing reagents, such as TE-buffer or RNAlater was found to negatively influence DNA yield and quality, alter microbial diversity and proportions of certain taxa^12,33,34^. However, studies evaluating OMNIgene.GUT, a commercially available faeces collection/stabilization system, indicated this system was equivalent to, or superior in maintaining sample microbiota composition to freezing, resulting in very few alterations to fresh stool samples^12,15^. We found that stabilizing the faecal sample in OMNIgene.GUT tended to preserve *Proteobacteria*, a phylum which was depleted in frozen samples (Supplementary Fig. 6), as already observed^12^. OMNIgene.GUT favours isolation of *Prevotellaceae* family, but with no statistical significance (Fig. 6). Although we did find a significant increase in *Sutterella* genus in samples collected using OMNIgene.GUT (Supplementary Table 5), also documented by Choo *et al*.^12^, our findings suggest that microbiota composition in OMNIgene.GUT collected samples was comparable to freshly processed faeces.

The evaluated three DNA extraction kits successfully extracted bacterial DNA from faecal samples. MP kit was the most efficient, extracting approximately three times more DNA than MO BIO and QIA kits per mg faeces. The use of MP and QIA kits resulted in high quality of the extracted DNA, while MO BIO provided DNA of inferior quality, consistent with literature data^18,35–38^. Furthermore, DNA extraction from faecal samples collected using OMNIgene.GUT system resulted in higher DNA yield irrespective of the extraction kit utilized, thus decreasing the amount of faecal material required, as previously reported^15^. MP kit was also associated with higher richness of isolated taxa as observed in alpha diversity analysis (observed OTUs, Chao1 and PD_whole_tree) (Supplementary Fig. 3). This is probably due to its effective G+ cell wall disruption, resulting in extracting significantly more G+ bacteria including *Bifidobacteriaceae* (and *Actinobacteria* phylum) than the other two kits (Table 1, Supplementary Table 4). Statistical analysis indicated differences between MP and MO BIO kits, with MO BIO depleting *Lachnospiraceae*, the most abundant G+ family, as well as other G+ organisms from *Firmicutes* and *Actinobacteria* phyla. The largest effect sizes were observed for *Coriobacteriaceae*, *Streptococcaceae* and *Lachnospiraceae*. The discrepancy in extracting efficiency of G+ organisms (illustrated in Supplementary Fig. 6) was previously suggested to relate to suboptimal lysing conditions in MO BIO extraction method^36^. The additional bead beating step proposed for MO BIO protocol^38^ was included in this study, but it did not improve the G+ recovery as was expected. When QIA was compared to other kits, Wilcoxon rank test on simulated data did not detect significant differences and only two significant families were identified using the Kruskal Wallis test (Table 1). This could be a consequence of intrinsic variability among QIA extracted samples with OMNIgene.GUT collection system (Fig. 6) having an influence on the G+/G- relative abundance ratio in these samples (Supplemental Fig. 6). Although the recently published study aiming to standardise extraction procedures^38^ recommended QIA based protocol for faecal DNA extraction (protocol Q), the results of the MP protocol from the same study (protocol #14) demonstrated high DNA quantity and quality, as well as community diversity and G+ recovery, positioning this extraction method high on the performance scale.

The influence of using different NGS platforms, MiSeq and IT, on microbial community composition analyses was already compared using different experimental setups and conditions^7,9,26,27^. While most of the published work assessed the performance of NGS approaches using the same primer set(s) across platforms, this study focused on investigating the comparability of taxonomic composition obtained with supplier recommended sequencing protocols, including the 16S rRNA gene regions and suggested primers.

The performance of platforms varied with respect to annotation levels. MiSeq annotation slightly outperformed IT at lower taxonomy levels since higher proportions of the assigned OTUs were obtained at family, genus and species levels (Fig. 3). This could be attributed to several platform set-up related factors, including differences in sequencing depth, targeted 16S gene region, amplicon sequencing lengths and/or single vs. pair-end sequencing technology^7,9,27^. Additionally, the mock community revealed that both sequencing technologies have the ability to detect all bacteria present in the mock sample with >99% of detected OTUs annotated correctly down to family level. In contrast to previous platform comparison using even and uneven mock community designed for that study^9^, we identified all 17 genera present in our mock community sample (HM-782D). The detected false positive genera comprised <1% of total OTU counts, resulting in overall substantial correspondence to theoretical composition unlike the abovementioned study. The different capacity of platforms to capture mock community diversity in these two studies could be related to the differences in community composition, tested 16S gene region and primer sets used. The relative abundances of taxa in our study were not fully reproduced and MiSeq in general had closer proportions and improved detection of *Actinobacteria* (*Actinomycetaceae* (*Actinomyces odontolyticus*) and *Propionibacteriaceae* (*Propionibacterium acnes*)), when compared to the expected values. This is in accordance with previous work on the same mock sample^7^ that also found marked differences with these organisms and attributed them to premature read truncation observed on the IT platform. In general, we found more deviations from theoretical abundance with IT than with MiSeq sequencing of mock community (Supplementary Fig. 2).

Since defined mock community lacks the complexity of the human faecal microbiota, further evaluation included comparison of human samples with respect to total number of commonly annotated taxa and detected diversity on two platforms. Alpha diversity indices demonstrated tendency of MiSeq technology to detect more organisms in the samples than IT but the phylogenetic distance of these taxa was similar (Fig. 4b). Additionally, the major driver of diversity between samples, presented as the beta diversity, was the platform used for the sequencing step (PCoA1: 22.7% variance), followed by the donor (PCoAs2–4 explaining 22.1%, 12.3% and 9.8% of variance), totalling 66.9% of total variance between samples. No major trends related to  kit, storage or extraction method were observed.

The assessment of relative abundances did not reveal marked differences between platforms (Supplementary Fig. 4). However, the statistical analyses taking into consideration the compositional nature of the NGS data and using Kruskal Wallis test on clr transformed dataset demonstrated statistical differences at all taxonomic levels, showed numerous taxa in this study to be exposed to technology driven bias (Supplementary Tables 3 and 4). However, as the level of significance does not by itself predict the magnitude of the differences further evaluations were performed. Primarily, to ensure that false positives (type I error) are avoided and to account for the technical variation and imprecision intrinsic to NGS datasets, Monte-Carlo simulations from Dirichlet distribution were generated on the original data followed by the evaluation of the effect sizes and additional parametric and non-parametric statistics. Statistical significance (Kruskal Wallis) revealed a substantial number of taxa (ten on family level) apparently guiding the platform difference (Supplementary Tables 3 and 4). After accounting for technical variation (Wilcoxon) the number declined (eight families remained). Scrutinizing for the effect size and filtering out only the taxa with medium to large effect sizes^39^ the number was further reduced (four families with effect size >0.5). We considered these taxa as the most relevant drivers of platform differences. The notable finding of this study, and not reported so far, was the increased capacity of MiSeq to detect *Verrucomicrobia* (*Akkermansia muciniphila* species was the only representative of this phylum) as well as *Faecalibacterium* genus with abundances being 10 and 5 fold higher than with IT, respectively. This finding may be of special interest to researchers of health promoting bacteria since both *Faecalibacterium prausnitzii*, a butyrate-producing species in the *Ruminococcaceae* family, and *Akkermansia muciniphila*, a mucus-colonizing and degrading bacteria, showed sufficient efficacies to be considered next generation probiotics^40^. Other taxa of interest that were statistically significant and 2 fold more abundant on MiSeq were [*Odoribacteraceae*], *Erysipelotrichaceae* and *Ruminococcaceae* (Supplementary Table 3). Additionally, the property that also separated MiSeq from IT was its improved consistency in detecting organisms across samples (fitness to linear model) and lower error margins (Fig. 7c) which could potentially be attributed to the fact that MiSeq pair-end sequencing produces longer reads versus single-ended IT (mean length ± SD - amplicon 451 ± 14 bp and read 213 ± 17 bp, respectively). Although previous reports suggested that the sequencing approach of using more than one primer pair covering more 16S rRNA gene regions should better represent the content of a complex bacterial sample^9,41^, we failed to see this improvement with experimental setup here described. The higher overall richness in MiSeq in this study could be attributed to the higher average sequencing depth (2.3 fold in MiSeq) or to the lower required matching sequence for MiSeq (V3-V4 region) to assign taxonomy to an OTU, than for IT (V2-V4, V6-V9).

In conclusion, OMNIgene.GUT was found to enable reliable faecal sample collection, transport and storage (comparable to freshly collected material) with respect to DNA amount and quality, except when QIA kit was subsequently used for DNA extraction. Out of three kits used, MP provided most efficient DNA extraction resulting in significantly higher quantity and quality of the DNA, as well as higher richness of faecal microbiota content. Despite the fact that this study investigated optimized NGS platform technologies which differ in multiple parameters (16S rRNA gene region(s), primers, amplicon lengths, sequencing technology (single vs. pair-end)), when analysed using the same processing pipeline, both MiSeq and IT adequately reproduce donor faecal microbiota specificity. However, all the technology-related bias described here suggests that particular care should be given to appropriate methodology choices suitable for the microbial profiling in a specific study.

## Materials and Methods

### Study design

The outline of the study design is presented in Fig. 1. Subjects participating were healthy adults able to give their consent (n = 4, 2 males and 2 females, age range 33–42) who donated a single faeces sample that was collected in its fresh, native state (native) or using OMNIgene.GUT system (Omni). Aliquots of both initial samples were processed further immediately on day 0 using DNA isolation kits from MP Biomedicals (MP), Qiagen (QIA) and MO BIO (MO BIO). The remaining quantities were stored at −20 °C and room temperature for native and Omni sample, respectively. DNA isolation was repeated at day 14 using all three kits, thus yielding 12 DNA samples per single faeces sample. All DNA samples were subjected to 16 S rRNA gene amplicon sequencing on two NGS platforms – Illumina MiSeq and Ion Torrent PGM. A mock bacterial community sample, was used as a control sample of NGS technologies.

### Microbial mock community

Genomic DNA from Microbial Mock Community B (Even, Low Concentration), v5.1 L, for 16S rRNA Gene Sequencing (#HM-782D) was acquired from BEI Resources. It is comprised of DNA from 20 bacterial strains containing equimolar ribosomal RNA operon counts (100.000 copies per organism per μL) and the complete list of organisms can be found in Supplementary Table 1.

### Collection and storage

Informed consent was obtained from all participants and all procedures were in accordance with the approved study protocol (University of Zagreb School of Medicine Ethics Committee, case number 380-59-10106-14-55/149). A fraction of faeces sample (1–2 g) was collected fresh in its native state in a screw-cap sterile container. An aliquot was processed within a few hours of collection while the remaining aliquot was frozen at −20 °C for 14 days and processed after thawing. The other fraction of the same faeces sample (400–500 mg) was collected in OMNIgene.GUT kit (DNA Genotek Inc, Ottawa, Canada), a system for self-collection and liquid stabilization of microbial DNA from faeces for gut microbiome profiling, according to manufacturer’s protocol. Briefly, the faeces sample was thoroughly mixed with 2 mL of stabilization liquid in the provided tube. Again, two aliquots of this faeces sample were processed, one within a few hours of collection and the other at day 14, after two-week storage at room temperature.

### DNA extraction

Three commercial faecal DNA extraction kits were evaluated in this study: Power Fecal DNA Isolation Kit (MO BIO, #12830-50), QIAamp Fast DNA Stool Mini Kit (QIAGEN, #51604) and Fast DNA SPIN Kit for Feces (MP Biomedicals, #116570200). The manufacturers’ protocols were followed for each kit, and recommendations for application to hard-to-lyse organisms were incorporated (detailed protocols for each DNA extraction procedure are supplied in Supplementary Methods). All extractions were performed by the same experimenter. The sample size for DNA extraction from native faeces ranged from 50–250 mg, while for the OMNIgene.GUT system 250 µL was used. Prior to extraction, samples were homogenized by bead-beating^42^. Homogenization of samples was performed using Minilys homogenizer (Bertin Corp) in tubes prefilled with high quality beads which ensures disruption of the cell walls and release of the DNA molecules in solution. MP kit contains tubes with beads (Lysing matrix E) and for QIA and MO BIO kit homogenization was performed using Soil grinding kit SK38 (Bertin Pharma, #D34016), with 0.1 mm glass beads, 1.4 mm ceramic beads and 1 glass bead of 4.0 mm. After extraction, DNA integrity was assessed by 1% agarose gel electrophoresis.

### DNA quantitation

The DNA quantity and quality measurements were performed on two spectrophotometric devices, Nanodrop 2000 and Qubit 3.0 (Thermo Fisher Scientific), based on the absorbance and fluorescence readouts, respectively. The A_260_/A_280_ absorbance ratio determined with Nanodrop was used to estimate the quality and purity of the extracted DNA while concentration was measured with Qubit 3.0.

DNA concentration and absorbance ratio at A_260_/A_280_ are reported as mean values ± SEM (16 samples per group). P-values are reported after applying Bonferroni correction on unpaired two-sided t-test, p < 0.001 is reported as ***.

### DNA sequencing and post-processing

Faecal bacterial communities were profiled by 16S rRNA amplicon sequencing using two NGS platforms: Illumina MiSeq (MiSeq) and Ion Torrent PGM (IT). Manufacturer recommended primers, reagents and protocols were applied.

For MiSeq sequencing Nextera XT DNA Sample Preparation Kit (Illumina, #FC-131-1096) was used for construction of 16S libraries. Hypervariable regions V3-V4 of 16S rRNA gene were amplified using primers with overhanging adapter sequences for compatibility with Illumina index and sequencing adapters needed for paired-end sequencing, resulting in a single amplicon with mean length of 464 bp^43^. After PCR amplification and PCR product clean up with Agencourt AMPure XP (Beckman Coulter, #A63881), Illumina sequencing adapters and dual-index barcodes were added to the amplicon target with Nextera XT Index Kit (Illumina, #FC-131-1002). Sequencing was performed on MiSeq platform (Illumina) with MiSeq Reagent Kit v3 (Illumina, #MS-102-3003), according to the manufacturer’s instructions.

For IT sequencing 16S libraries from faecal DNA samples were constructed by amplifying seven regions V2, V4, V8 and V3, V6-V7, V9 of bacterial 16S rRNA using primers from Ion 16S™ Metagenomics Kit (Thermo Fisher Scientific, #A26216). Amplicons were further processed using manufacturer recommended protocols and ION PGM™ Template OT2 400 Kit (Thermo Fisher Scientific, #4479878). Final DNA library concentration was quantified using Qubit dsDNA BR Assay Kit (Thermo Fisher Scientific, #Q32850). Following library quantification the Ion OneTouch™ 2 (Thermo Fisher Scientific, #4474778) system was used to prepare template positive ion sphere particles containing the clonally amplified DNA libraries using the ION PGM™ Template OT2 400 Kit. Template positive sphere density was within the optimal range of 10–30%. Sequencing was performed on Ion Personal Genome Machine (PGM) System (Thermo Fisher Scientific) with Ion PGM Hi-Q Sequencing Kit (Thermo Fisher Scientific, #A25592) and Ion 316 Chip Kit v2 (Thermo Fisher Scientific, #4483188) according to the manufacturer’s instructions.

Raw sequencing files from both platforms were processed using QIIME pipeline^44^. For MiSeq, fastq files containing paired end reads were first merged, allowing overlap between mates (‘–allow-outies’ option), using FLASh^45^. Then for both platforms,.fastq files were trimmed, filtered by quality and chimera removed as described in the default QIIME pipeline. Operational Taxonomic Units (OTUs) were assigned using the vsearch^46^ algorithm and PyNast alignment^47^ against the GreenGenes database (version 13_8, May 2013). Cumulative OTU counts for each taxonomy level from phylum to genus were extracted into separate tables from the resulting assignment.

Subsequent processing and analysis was done for each taxonomy level separately according to the Gloor CoDa Microbiome tutorial^48^, using ALDEx2 R package^30^ with additions of multivariate generalized linear models for testing the significance of multiple factors and their interactions.

## Data analysis

### Alpha and beta diversity analyses

The compositional diversity within each sample was ascertained using several alpha diversity indices: observed OTUs, followed by the PD whole tree and Chao1, as implemented in the QIIME pipeline. Sequences were rarefied from 0 to 30 000 range. Beta diversity was reported using weighted UniFrac distance measure^49^, and major contributions to the differences in beta diversity were presented as principle coordinate analysis, as implemented in the QIIME pipeline.

### Precision analysis

To assess the reproducibility between relative taxa abundances across time points and collection/storage parameters within a DNA extraction kit, abundance values were plotted against reference point (native 0) abundance from the same kit. Linear regression analysis was used to plot the linear regression line and determine R^2^ - the goodness of fit of the line to the data.

Aggregate relative abundances at different taxonomic ranks as well as precision analysis were displayed on raw counts.

### OTU count normalisation and statistical analysis

The obtained sequencing data were evaluated as compositional data^50^ and the statistical analyses were performed as previously described^28–30^. Briefly, the approach incorporates Bayesian multiplicative replacement of count zeros and centred log-ratio (clr) OTU count data transformation^30^. The centred log-ratio (clr) transformation^28^ ensures the data are scalable and sub-compositionally coherent thus making them amenable for both univariate and multivariate analyses. The raw OTU counts on taxonomic levels from phylum to genus were transformed to compositional values using the ALDEx2 (ANOVA-like differential expression analysis) R package.

Principal component analysis (PCA) is shown on zero-imputed and clr transformed counts using ‘prcomp’ function in R.

Kruskal-Wallis multivariate test was performed to detect overall significance for each variable per OTU. P-values were considered significant if p < 0.05 after Benjamini-Hochberg (BH) correction.

The transformed data were further assessed for significant differences among the group of samples collected/processed using Wilcoxon rank test and Welch’s t-test, as implemented in ALDEx2 pipeline. Tests were performed on 50 Monte Carlo simulated instances of Dirichlet-distributed original datasets to estimate per-feature technical variation within each sample. All p-values are reported after adjusting for multiple testing with BH correction. Pairwise comparisons of transformed data were performed for pairs of donors, time points, kits and faecal collection type for each taxonomic level (phylum to genus).

## Electronic supplementary material

Supplementary information
