# Supplementary material for: Methodology challenges in studying human gut microbiota – effects of collection, storage, DNA extraction and next generation sequencing technologies
Source: Sci Rep. 2018 Mar 23;8:5143. doi: 10.1038/s41598-018-23296-4 (PMC5865204; doi:10.1038/s41598-018-23296-4)
Supplement: Supplementary file 1 — Supplementary information [file 41598_2018_23296_MOESM1_ESM.pdf]

Methodology challenges in studying human gut microbiota – effects of collection, storage, DNA extraction and next generation sequencing technologies

Marina Panek[1*], Hana Čipčić-Paljetak[1*], Anja Barešić[2*], Mihaela Perić[1], Mario Matijašić[1], Ivana Lojkić[3], Darija Vranešić Bender[4], Željko Krznarić[5], Donatella Verbanac[1]

[1] University of Zagreb School of Medicine, Center for Translational and Clinical Research, Šalata 2, 10000 Zagreb, Croatia

[2] MRC London Institute of Medical Sciences, Du Cane Road, London, W12 0NN, UK

[3] Croatian Veterinary Institute, Department for Virology, Savska cesta 143, 10000 Zagreb, Croatia

[4] University Hospital Zagreb, Department of Internal Medicine, Unit of Clinical Nutrition, Kišpatićeva 12, 10000 Zagreb, Croatia

[5] University of Zagreb School of Medicine, Department of Internal Medicine, Kišpatićeva 12, 10000 Zagreb, Croatia

Supplementary Table 1. Microbial mock community #HM-782D

Organism	NCBI reference sequence
Acinetobacter baumannii, 5377	NC_009085
Actinomyces odontolyticus, 1A.21	NZ_AAYI02000000
Bacillus cereus, NRS 248	NC_003909
Bacteroides vulgatus, ATCC 8482	NC_009614
Clostridium beijerinckii, NCIMB 8052	NC_009617
Deinococcus radiodurans, R1 smooth	NC_001263, NC_001264
Enterococcus faecalis, OG1RF	NC_17316
Escherichia coli, K12, substrain MG1655	NC_000913
Helicobacter pylori, 26695	NC_000915
Lactobacillus gasseri, 63 AM	NC_008530
Listeria monocytogenes, EGDe	NC_003210
Neisseria meningitidis, MC58	NC_003112
Propionibacterium acnes, KPA171202	NC_006085
Pseudomonas aeruginosa, PAO1-LAC	NC_002516
Rhodobacter sphaeroides, ATH 2.4.1	NC_007493, NC_007494
Staphylococcus aureus, TCH1516	NC_010079
Staphylococcus epidermidis, PCI 1200	NC_004461
Streptococcus agalactiae, 2603 V/R	NC_004116
Streptococcus mutans, UA159	NC_004350
Streptococcus pneumoniae, TIGR4	NC_003028

Supplementary Table 2. Overview of kit associated cell lysis method, sample size and obtained DNA concentrations for studied DNA extraction kits.

DNA extraction kit	method of cell lysis	faeces sample	sample size	DNA day 0* ng/µL	DNA day 14* ng/µL
MO BIO<br>Power Fecal DNA Isolation Kit	beads<br>lysis solution<br>heat	native	250 mg	10.9±1.9	11.5±3.5
		Omnigene	250 µL	17.1±5.3	5.7±0.8
MP Biomedicals<br>Fast DNA SPIN Kit for Feces	beads<br>lysis solution	native	50 mg	99.2±16.0	98.6±24.3
		Omnigene	250 µL	96.2±22.8	75.9±18.4
QIAGEN<br>QIAamp Fast DNA Stool Mini Kit	beads<br>lysis solution<br>heat	native	200 mg	46.0±9.1	52.5±11.2
		Omnigene	250 µL	30.6±13.2	46.3±23.4

* mean value of all DNA sample measurements ±SEM

Supplementary Table 3. Taxon significance between platforms and kits. Benjamini Hochberg corrected p-values less than 0,05 (except effect columns) obtained using Kruskal-Wallis test with taxa stratified to pairs of values for each variable, and using Wilcoxon test on 50 Monte Carlo dataset replicates for each combination of variables. Tests performed on clr (centered log ratio) normalized OTU count. Effect represents effect size i.e. the median difference between groups divided by the largest median variation within groups.

	Kruskal-Wallis				50 MC Wilcoxon					
	IL:IT	MOBIO:MP	MOBIO:QIA	MP:QIA	IL:IT	effect*	MOBIO:other	effect*	MP:other	effect*
**phylum**										
Actinobacteria		0,000			0,040	0,336	0,004	0,602	0,001	0,621
Firmicutes	0,039	0,000		0,035					0,018	0,426
Lentisphaerae	0,009				0,037	-0,297				
Proteobacteria					0,011	0,434				
Tenericutes	0,044				0,044	0,299				
Verrucomicrobia	0,000				0,000	-0,616				
**class**										
Actinobacteria		0,005							0,036	0,358
Bacilli		0,000	0,043	0,029	0,028	0,387	0,000	0,801	0,000	0,832
Bacteroidia	0,023								0,016	0,402
Clostridia	0,003	0,000							0,000	0,661
Coriobacteriia		0,000	0,043				0,000	0,695		
Deltaproteobacteria	0,001	0,026								
Erysipelotrichi	0,001	0,029								
Verrucomicrobiae	0,000				0,000	-0,658				
**order**										
Bacteroidales	0,001									
Bifidobacteriales		0,007							0,036	0,344
Clostridiales	0,000	0,001							0,023	0,437
Coriobacteriales		0,000	0,041				0,000	0,694	0,000	0,736
Desulfovibrionales	0,000	0,030								
Erysipelotrichales	0,000	0,034			0,028	-0,394			0,000	0,881
Lactobacillales		0,000	0,041	0,025			0,000	0,819		
Verrucomicrobiales	0,000				0,000	-0,710				
Victivallales	0,002									
**family**										
[Mogibacteriaceae]		0,000		0,023			0,045	0,445	0,002	0,641
[Odoribacteraceae]	0,000				0,000	-0,610				
Bacteroidaceae	0,001									
Bifidobacteriaceae		0,003							0,016	0,348
Clostridiaceae		0,000							0,010	0,526
Coriobacteriaceae		0,000					0,000	0,733	0,001	0,666
Desulfovibrionaceae	0,000				0,015	-0,395				
Erysipelotrichaceae	0,000				0,002	-0,540				
Lachnospiraceae	0,019	0,000		0,023					0,001	0,635
Peptostreptococcaceae		0,000							0,001	0,559
Porphyromonadaceae	0,000				0,028	-0,424				
Ruminococcaceae	0,000				0,001	-0,557				
Streptococcaceae		0,000					0,000	0,755	0,001	0,674
Veillonellaceae	0,000				0,014	-0,413				
Verrucomicrobiaceae	0,000				0,000	-0,769				
Victivallaceae	0,001				0,007	-0,451				
**genus**										

[Ruminococcus]		0,004		0,039					0,016	0,484
Adlercreutzia		0,000	0,012				0,000	0,797	0,000	0,706
Akkermansia	0,000				0,000	-0,773				
Anaerostipes	0,005				0,013	-0,252				
Bacteroides	0,001				0,036	-0,379				
Bifidobacterium		0,004							0,032	0,277
Bilophila	0,000				0,003	-0,519				
Blautia	0,011	0,000		0,002			0,000	0,705	0,000	0,930
Clostridium	0,005	0,000			0,002	0,536			0,011	0,490
Coprococcus	0,009						0,025	0,527	0,000	0,684
Dorea		0,000							0,005	0,600
Faecalibacterium	0,000				0,000	-1,135				
Lachnospira	0,012									
Odoribacter	0,000				0,000	-0,797				
Oscillospira	0,005									
Parabacteroides	0,000				0,100	-0,454				
Paraprevotella	0,044									
Phascolarctobacterium	0,003				0,024	-0,364				
Roseburia	0,002				0,012	-0,425				
Ruminococcus	0,045									
SMB53	0,031	0,000		0,039					0,002	0,541
Streptococcus		0,000					0,001	0,603	0,001	0,593

* >±0,5 indicates relevance

Supplementary Table 4: Taxon significance between platforms and kits. Test for significant factors and their interactions for each taxon at specific level. Tests were performed on 50 Monte Carlo samples of Dirichlet-distributed original datasets. Generalized linear model was built for each OTU as response and by taking into account first and second order interactions between covariates. ANOVA log-likelihood ratio test was used to determine significant factors and interactions. P-values were averaged across Monte Carlo instances and corrected using Benjamini-Hochberg method.

**A.** IT vs.IL	**rab.all**	**rab.win.IL**	**rab.win.IT**	**diff.btw**	**diff.win**	**effect**	**overlap**	**we.ep**	**we.eBH**	**wi.ep**	**wi.eBH**
**phylum**											
Actinobacteria	4.379	3.860	4.930	1.187	3.300	0.336	0.358	0.012	0.032	0.020	0.040
Lentisphaerae	-3.406	-2.741	-4.135	-1.553	4.966	-0.297	0.353	0.040	0.070	0.020	0.037
Proteobacteria	7.265	6.367	7.920	1.389	2.971	0.434	0.313	0.004	0.019	0.003	0.011
Tenericutes	-2.560	-2.960	-1.193	2.259	7.280	0.299	0.356	0.053	0.089	0.025	0.044
Verrucomicrobia	-4.757	-3.125	-6.148	-3.321	4.842	-0.616	0.232	<0.001	0.001	<0.001	<0.001
**class**											
[Lentisphaeria]	-1.449	-0.478	-2.598	-1.988	4.859	-0.356	0.330	0.013	0.049	0.004	0.025
Bacilli	5.283	4.814	5.861	1.063	2.676	0.387	0.327	0.006	0.041	0.004	0.028
Verrucomicrobiae	-3.119	-1.079	-4.712	-3.867	5.349	-0.658	0.218	<0.001	<0.001	<0.001	<0.001
**order**											
Erysipelotrichales	7.426	7.948	6.857	-0.931	2.240	-0.394	0.323	0.004	0.032	0.004	0.028
Flavobacteriales	-2.497	-1.332	-3.426	-1.997	4.296	-0.410	0.306	0.038	0.110	0.006	0.031
Fusobacteriales	-2.102	-0.859	-3.270	-2.320	4.332	-0.490	0.281	0.006	0.027	0.002	0.009
Verrucomicrobiales	-1.340	0.946	-2.932	-4.116	5.488	-0.710	0.208	<0.001	<0.001	<0.001	<0.001
Victivallales	0.534	1.889	-0.587	-2.412	4.979	-0.451	0.293	0.007	0.045	0.001	0.011
**family**											
[Odoribacteraceae]	8.393	8.873	7.747	-1.215	1.797	-0.603	0.236	<0.001	0.001	<0.001	<0.001

Desulfovibrionaceae	5.766	6.254	5.300	-0.937	1.973	-0.395	0.316	0.034	0.116	0.002	0.015
Erysipelotrichaceae	7.524	8.182	6.923	-1.153	2.044	-0.540	0.265	<0.001	0.003	<0.001	0.002
Flavobacteriaceae	-2.477	-1.307	-3.392	-2.094	4.268	-0.421	0.295	0.030	0.093	0.005	0.029
Fusobacteriaceae	-1.946	-0.522	-3.039	-2.457	4.347	-0.535	0.261	0.003	0.020	<0.001	0.003
Porphyromonadaceae	9.629	9.918	9.160	-1.077	2.310	-0.424	0.328	0.002	0.017	0.004	0.028
Ruminococcaceae	12.578	13.111	11.909	-1.052	1.713	-0.557	0.266	<0.001	0.004	<0.001	0.001
Veillonellaceae	9.316	9.710	8.977	-0.726	1.515	-0.413	0.309	0.008	0.053	0.002	0.014
Verrucomicrobiaceae	-1.244	1.287	-2.915	-4.598	5.586	-0.769	0.201	<0.001	<0.001	<0.001	<0.001
Victivallaceae	0.511	2.001	-0.729	-2.580	5.248	-0.451	0.296	0.002	0.018	0.001	0.007
**genus**											
Akkermansia	-0.251	2.390	-1.811	-4.518	5.514	-0.773	0.208	<0.001	<0.001	<0.001	<0.001
Anaerostipes	5.395	5.980	4.505	-1.525	5.865	-0.252	0.309	0.145	0.344	0.001	0.013
Bacteroides	15.022	15.322	14.625	-0.812	2.037	-0.379	0.336	0.002	0.020	0.005	0.036
Bilophila	6.918	7.507	6.324	-1.066	1.815	-0.519	0.277	0.020	0.079	<0.001	0.003
Clostridium	6.392	5.688	6.873	1.252	2.127	0.536	0.276	<0.001	0.001	<0.001	0.002
Eggerthella	1.886	2.680	0.694	-2.173	4.702	-0.398	0.311	0.003	0.027	0.001	0.014
Faecalibacterium	11.415	12.236	9.845	-2.257	1.726	-1.135	0.125	<0.001	<0.001	<0.001	<0.001
Fusobacterium	-0.947	0.547	-2.116	-2.466	4.486	-0.495	0.264	0.003	0.024	<0.001	0.004
Odoribacter	9.072	9.850	8.162	-1.716	1.960	-0.797	0.162	<0.001	<0.001	<0.001	<0.001
Parabacteroides	10.836	11.143	10.302	-1.120	2.261	-0.454	0.303	<0.001	0.007	0.001	0.010
Phascolarctobacterium	9.795	10.329	9.458	-0.900	2.478	-0.364	0.325	0.007	0.056	0.003	0.024
Roseburia	9.317	9.765	8.824	-0.931	2.016	-0.425	0.303	0.001	0.016	0.001	0.012
Shuttleworthia	-1.818	-2.655	-0.542	2.221	4.540	0.426	0.289	0.012	0.056	0.004	0.023
Veillonella	2.048	3.037	1.417	-1.574	4.229	-0.340	0.335	0.014	0.081	0.006	0.042
**B.** Others vs. MO BIO	**rab.all**	**rab.win. MO.BIO**	**rab.win. Others**	**diff.btw**	**diff.win**	**effect**	**overlap**	**we.ep**	**we.eBH**	**wi.ep**	**wi.eBH**
**phylum**											
Actinobacteria	4.379	2.76	4.929	1.985	3.148	0.602	0.26	<0.001	0.002	<0.001	0.004
**class**											
Bacilli	5.283	3.695	5.794	2.087	2.446	0.801	0.186	<0.001	0.001	<0.001	<0.001
Coriobacteriia	5.164	3.733	5.763	1.859	2.563	0.695	0.235	<0.001	0.001	<0.001	<0.001
**order**											
Coriobacteriales	7.225	5.640	7.851	1.874	2.513	0.694	0.214	<0.001	0.002	<0.001	<0.001
Lactobacillales	7.294	5.743	7.865	2.051	2.309	0.819	0.190	<0.001	0.001	<0.001	<0.001
**family**											
[Mogibacteriaceae]	4.764	4.098	5.129	0.970	1.952	0.445	0.292	0.016	0.126	0.003	0.045
Coriobacteriaceae	7.333	5.883	8.087	1.939	2.470	0.733	0.206	<0.001	0.003	<0.001	<0.001
Streptococcaceae	6.493	5.685	7.155	1.862	2.356	0.755	0.199	<0.001	0.001	<0.001	<0.001
**genus**											
Adlercreutzia	4.632	3.185	5.481	2.436	2.688	0.797	0.160	<0.001	<0.001	<0.001	<0.001
Blautia	11.365	10.605	11.767	1.224	1.584	0.705	0.205	<0.001	0.002	<0.001	<0.001
Coprococcus	10.819	10.299	11.048	0.804	1.384	0.527	0.279	0.003	0.057	0.001	0.025
Streptococcus	7.573	6.825	8.179	1.667	2.531	0.603	0.228	<0.001	0.007	<0.001	0.001
**C.** MP vs. others	**rab.all**	**rab.win. Others**	**rab.win. MP**	**diff.btw**	**diff.win**	**effect**	**overlap**	**we.ep**	**we.eBH**	**wi.ep**	**wi.eBH**
**phylum**											
Actinobacteria	4.379	3.785	5.876	2.050	3.142	0.621	0.246	<0.001	<0.001	<0.001	0.001
Firmicutes	10.198	9.876	10.663	0.905	2.039	0.426	0.303	0.003	0.015	0.004	0.018
**class**											
Actinobacteria	5.102	4.303	6.600	2.029	5.053	0.358	0.331	0.014	0.060	0.007	0.036
Bacilli	5.283	4.489	6.254	2.041	2.328	0.832	0.186	<0.001	<0.001	<0.001	<0.001
Clostridia	11.999	11.766	12.382	0.599	1.382	0.402	0.306	0.002	0.011	0.003	0.016
Coriobacteriia	5.164	4.520	6.307	1.828	2.454	0.661	0.237	<0.001	<0.001	<0.001	<0.001
**order**											
Bifidobacteriales	7.196	6.388	8.765	2.105	5.632	0.344	0.315	0.013	0.086	0.004	0.036
Clostridiales	13.985	13.760	14.379	0.675	1.369	0.437	0.302	0.001	0.011	0.002	0.023
Coriobacteriales	7.225	6.506	8.326	1.900	2.348	0.736	0.228	<0.001	<0.001	<0.001	<0.001
Lactobacillales	7.294	6.356	8.371	2.044	2.283	0.881	0.165	<0.001	<0.001	<0.001	<0.001
**family**											
[Mogibacteriaceae]	4.764	4.338	5.667	1.234	1.839	0.641	0.252	<0.001	0.001	<0.001	0.002
Bifidobacteriaceae	7.448	6.631	8.903	1.918	6.044	0.348	0.293	0.022	0.164	0.002	0.016
Clostridiaceae	7.503	7.099	7.972	1.027	1.842	0.526	0.291	<0.001	0.003	0.001	0.010

Coriobacteriaceae	7.333	6.416	8.397	1.732	2.301	0.666	0.233	<0.001	<0.001	<0.001	0.001
Lachnospiraceae	12.861	12.620	13.439	0.920	1.398	0.635	0.253	<0.001	0.001	<0.001	0.001
Peptostreptococcaceae	5.072	4.629	5.983	1.420	2.211	0.559	0.241	<0.001	<0.001	<0.001	0.001
Streptococcaceae	6.493	5.996	7.864	1.701	2.321	0.674	0.223	<0.001	<0.001	<0.001	0.001
**genus**											
[Ruminococcus]	9.209	8.858	10.054	1.285	2.459	0.484	0.281	0.001	0.008	0.001	0.016
Adlercreutzia	4.632	3.781	5.707	2.060	2.901	0.706	0.205	<0.001	<0.001	<0.001	<0.001
Bifidobacterium	8.694	7.927	10.224	1.857	6.986	0.277	0.309	0.060	0.336	0.003	0.032
Blautia	11.365	10.936	12.470	1.536	1.538	0.930	0.149	<0.001	<0.001	<0.001	<0.001
Clostridium	6.392	5.773	6.958	1.104	1.954	0.490	0.277	<0.001	0.005	0.001	0.011
Coprococcus	10.819	10.499	11.426	0.967	1.317	0.684	0.213	<0.001	<0.001	<0.001	<0.001
Dorea	8.883	8.521	9.321	0.897	1.282	0.600	0.261	<0.001	0.001	<0.001	0.004
Eggerthella	1.886	1.146	3.509	2.531	4.585	0.508	0.266	0.005	0.043	0.001	0.008
SMB53	4.552	3.720	5.459	1.809	2.867	0.541	0.262	0.002	0.023	<0.001	0.002
Streptococcus	7.573	7.212	8.682	1.629	2.589	0.593	0.228	<0.001	<0.001	<0.001	0.001
**D.** Omni 0 vs. others	**rab.all**	**rab.win. Others**	**rab.win. Omni 0**	**diff.btw**	**diff.win**	**effect**	**overlap**	**we.ep**	**we.eBH**	**wi.ep**	**wi.eBH**
**class**											
Betaproteobacteria	7.068	6.784	8.160	1.189	2.086	0.533	0.288	0.002	0.042	0.002	0.035
**order**											
Burkholderiales	9.045	8.784	10.071	1.215	1.999	0.578	0.257	0.001	0.037	0.001	0.024
**family**											
Alcaligenaceae	9.188	8.822	10.003	1.302	1.765	0.702	0.206	<0.001	0.002	<0.001	0.004
**genus**											
Sutterella	10.283	9.885	11.198	1.342	1.743	0.68	0.22	<0.001	0.003	<0.001	0.004

rab.all - median clr value for all samples in the feature; rab.win.NS - median clr value for the NS group of samples; rab.win.S - median clr value for the S group of samples; dif.btw - median difference in clr values between S and NS groups; dif.win - median of the largest difference in clr values within S and NS groups; effect - median effect size: diff.btw / max(dif.win) for all instances; overlap - proportion of effect size that overlaps 0 (i.e. no effect); we.ep - Expected p-value of Welch's t-test; we.eBH - Expected Benjamini-Hochberg corrected p-value of Welch's t-test; wi.ep - Expected p-value of Wilcoxon rank test; wi.eBH - Expected Benjamini-Hochberg corrected p-value of Wilcoxon test

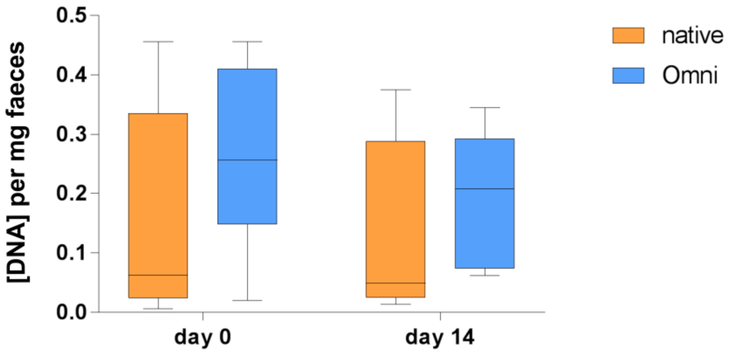

Supplementary Figure 1. Stabilized samples have increased DNA yield. DNA yield from native (native) and OMNIgene.GUT (Omni) samples at two different time points (n=12). DNA yield is expressed as DNA concentration (ng/µl) normalized by quantity of faeces used for extraction.

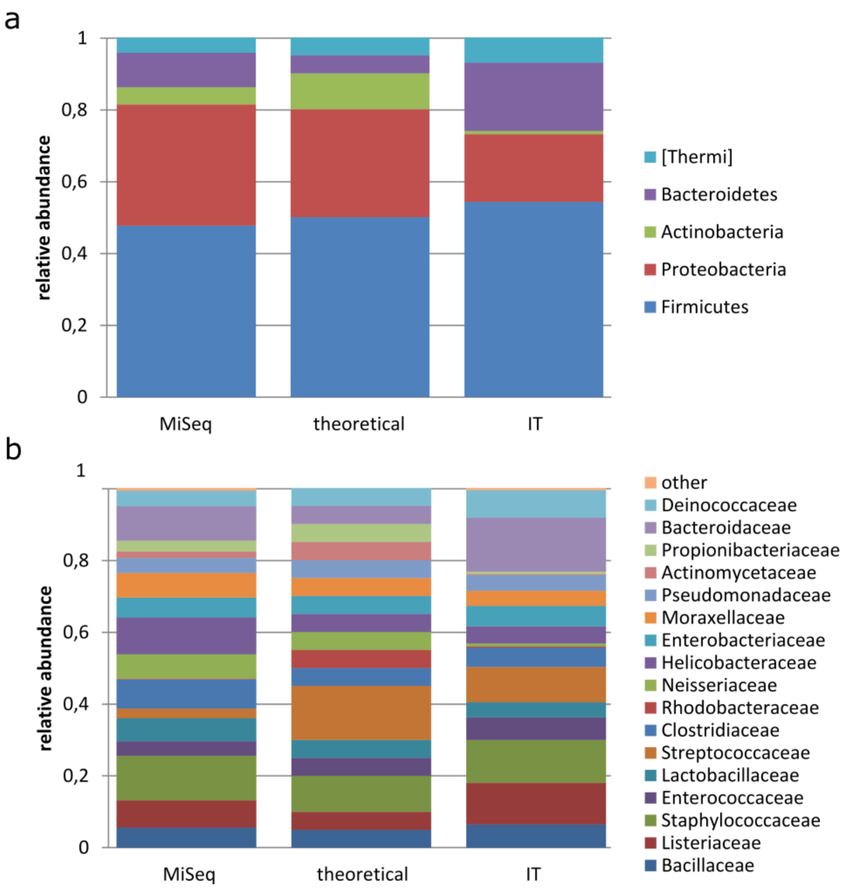

Supplementary Figure 2: Bacterial mock community. Comparison of bacterial mock sample sequenced on Illumina MiSeq (MiSeq) and Ion Torrent PGM (IT), with the theoretically expected values on a) phylum and b) family level.

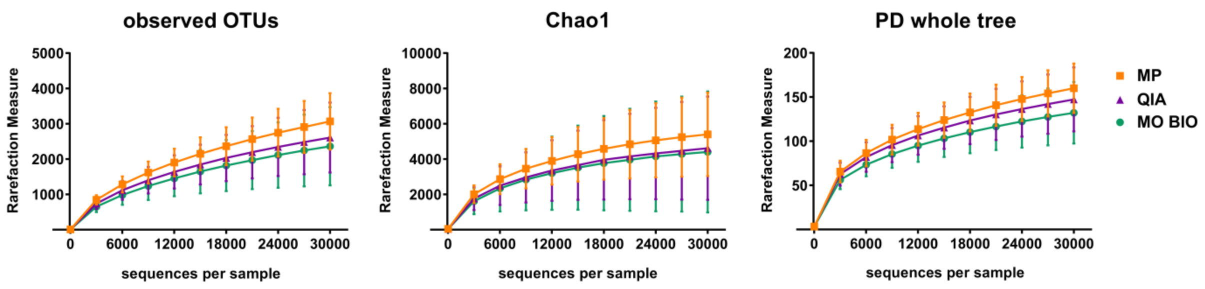

Supplementary Figure 3: Alpha diversity for each DNA extraction kit. Alpha diversity indices for a range of rarified sequence depths: observed OTUs, Chao1 index and PD_whole_tree index.

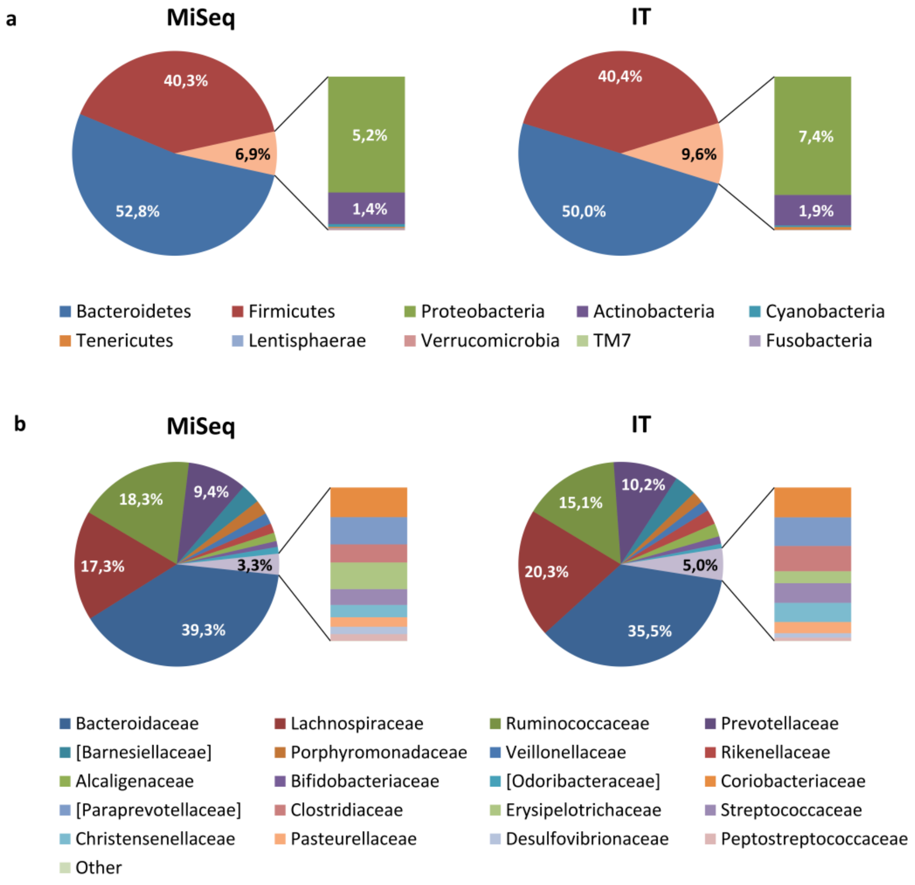

Supplementary Figure 4: Relative abundance of taxa per platform. Data represented at (a) phylum and (b) family level. (a) Phyla with <10% abundance are shown on a side bar. (b) 20 most abundant families are presented, the remainder have been summed as "Other". The 10 major families are displayed on pie chart, with the remaining ones are on a side bar.

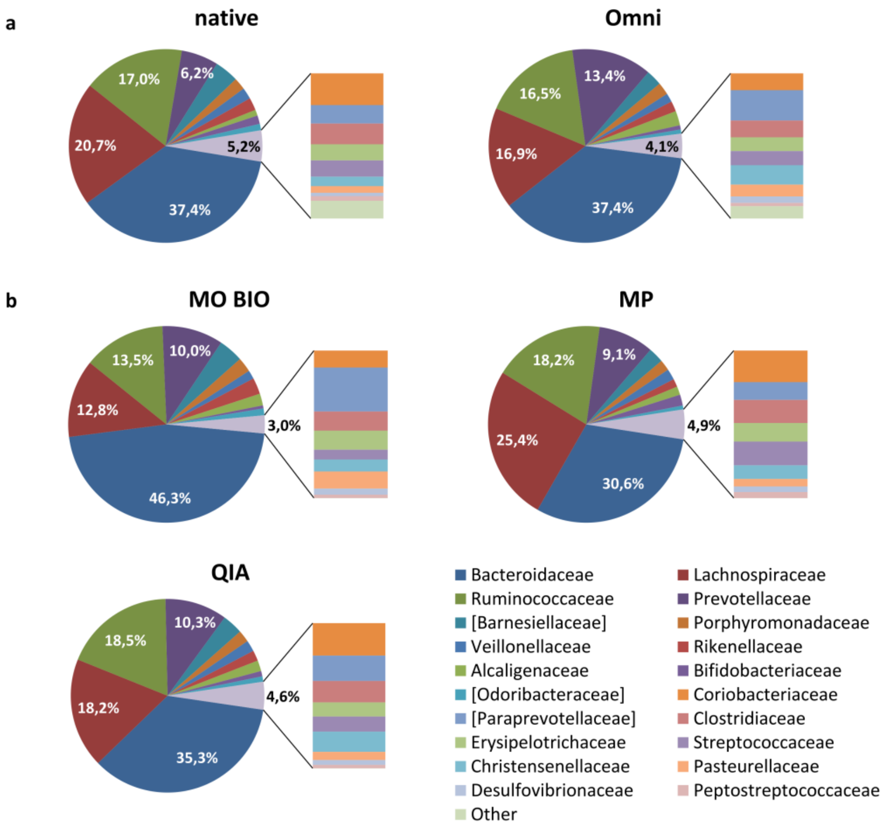

Supplementary Figure 5: Relative abundance data, divided by (a) sample stabilization procedure and (b) DNA extraction kits. Data represented at family level with 20 most abundant families are presented, the remainder have been summed as "Other". The 10 major families are displayed on pie chart, with the remaining ones on a side bar.

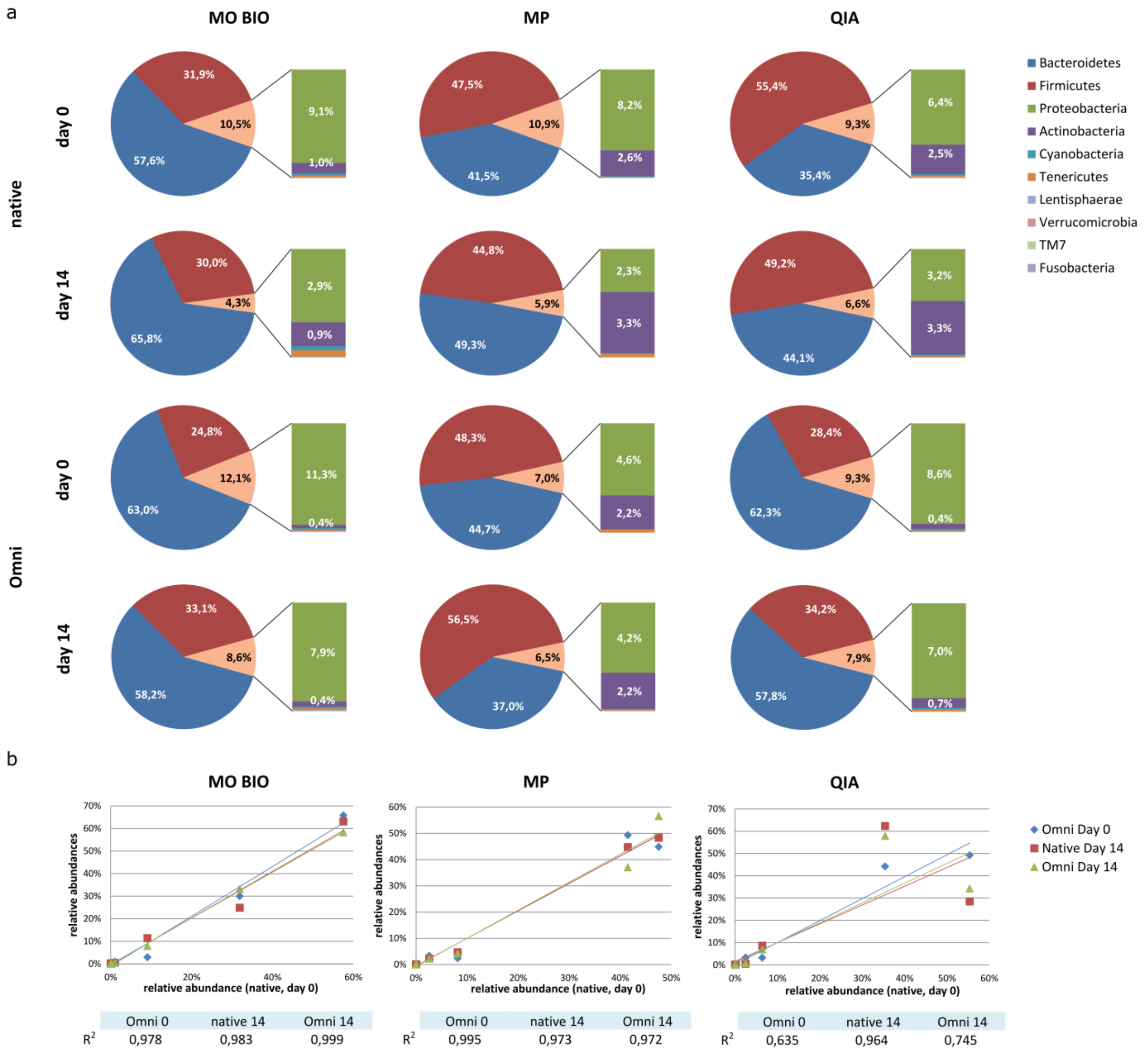

Supplementary Figure 6. **Relative abundance of phyla depends on sample collection/storage and DNA extraction procedure.** Analysis of (a) DNA extraction procedures (MO BIO, MP, QIA) with respect to sampling time point (day 0, day 14) and sample collection procedure (native, Omni). Phyla with <10% abundance are shown on a side bar. (b) Native 0 sample relative phyla abundances plotted against corresponding native 14, as well as Omni 0 and Omni 14 relative abundances for each kit. R^2 values for linear regression on each time point are displayed in the table below.

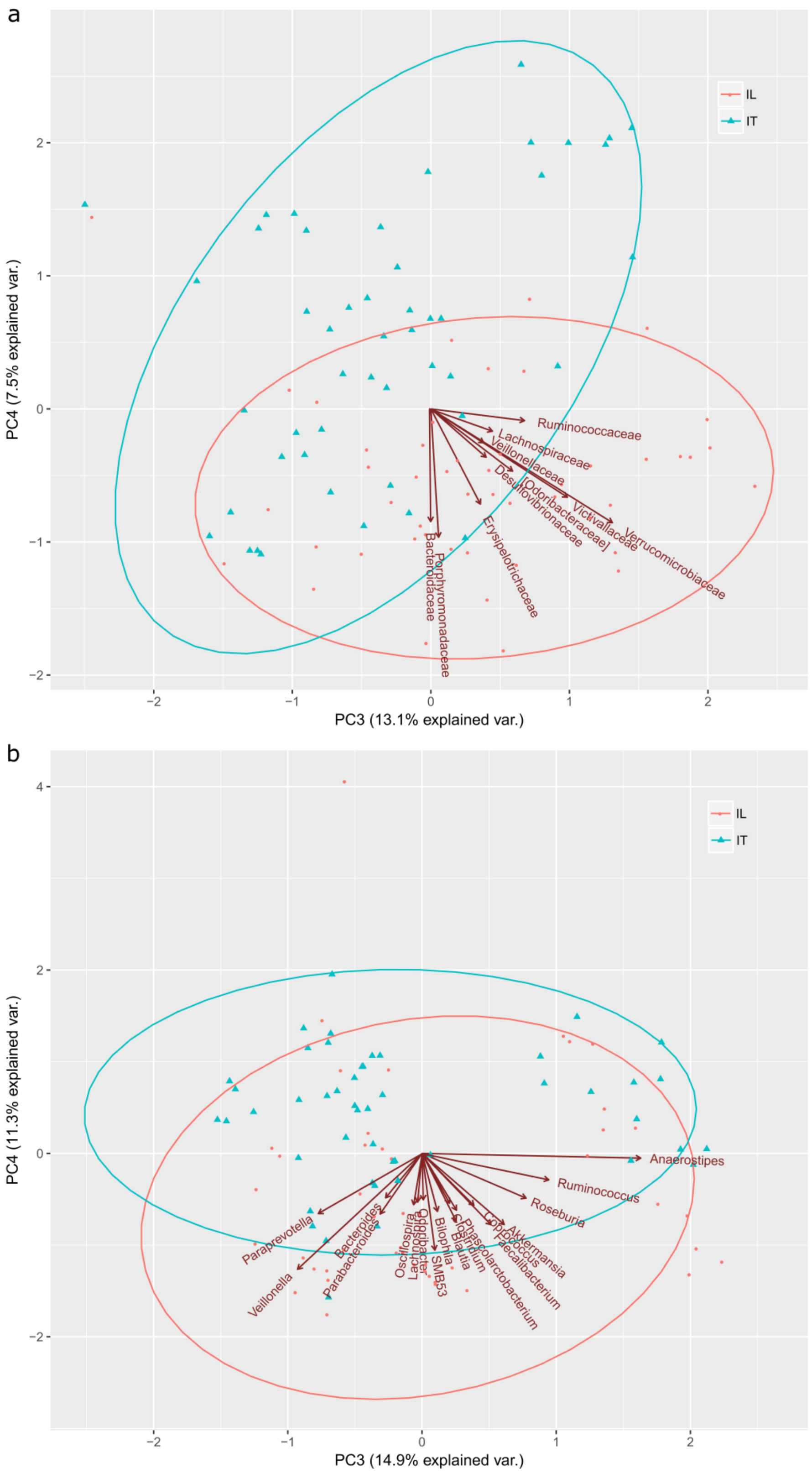

Supplementary Figure 7. **Platform differences are driven by specific taxa.** PCA bi-plots of clr transformed data on Illumina MiSeq and Ion Torrent PGM sequencing platforms on (a) family and (b) genus level. Only taxa with Benjamini-Hochberg adjusted p-values <0.05 in Kruskal-Wallis test are labelled.

Supplementary Methods. Detailed DNA extraction protocols.

Extraction of bacterial DNA from faecal samples using MP BIOMEDICAL Fast DNA spin kit for faeces (#116570200) according to the manufacturer's protocol:

- In a 2 mL Lysing Matrix E tube add 50 mg fresh faeces, or 250 µL OMNIgene.GUT faeces sample, 825 µL Sodium Phosphate Buffer and 275 µL of PLS solution. Shake to mix and then vortex 10-15 seconds.
- Centrifuge samples at 14,000 x g for 5 minutes and decant supernatant.
- Add 978 µL Sodium Phosphate Buffer and 12 µL MT Buffer. Vortex briefly to mix.
- Homogenize with Minilys homogenizer (Bertin Corp), speed 3 for 40 seconds.
- After homogenization, centrifuge samples at 14,000 x g for 10 minutes, transfer supernatant to a clean 2.0 ml centrifuge tube, add 250 µL of PPS solution and shake vigorously to mix.
- Incubate at 4˚C for 10 minutes. Do not vortex. Centrifuge samples at 14,000 x g for 2 minutes.
- Transfer supernatant to the Binding Matrix Solution in the 15 mL tube; shake gently by hand to mix for 3-5 minutes.
- Centrifuge samples at 14,000 x g for 2 minutes. Decant the supernatant.
- Wash the binding mixture pellet by gently resuspending with 1 mL Wash Buffer #1
- Transfer 600 µL of the binding mixture to a Spin filter tube and centrifuge at 14,000 x g for 1 minute. Empty the catch tube and add the remaining binding mixture to the Spin filter tube and centrifuge as before. Empty the catch tube again.
- Add 500 µl of wash buffer #2 to the spin filter tube and gently resuspend using the force of the liquid from the pipette tip to resuspend the pellet. Do not vortex.
- Centrifuge sample at 14,000 x g for 2 minute. Discard the flow-through.
- Centrifuge the sample again for 2 minutes to extract residual ethanol from the binding matrix and dry the sample.
- Transfer the spin filter bucket to a clean 1.9 ml catch tube. Add 100 µL TES and stir the matrix with a pipette tip to resuspend the pellet. Do not vortex.
- Centrifuge samples at 14,000 x g for 2 minutes to elute purified DNA into the clean catch tube. Discard the spin filter and store DNA sample at -20˚C.

Extraction of bacterial DNA from faecal samples using QIAamp DNA Stool Mini Kit (#51604) according to the manufacturer's protocol:

- In a 2 mL tube for homogenization (Soil grinding kit SK38) add 180-220 mg fresh faeces, or 250 µl OMNIgene.GUT faeces sample and mix with 1.4 mL of Buffer ASL.
- Homogenize with Minilys homogenizer (Bertin Corp), speed 2 for 40 seconds.
- After homogenization, heat the suspension for 5 minutes at 70˚C.
- Vortex sample for 15 seconds and centrifuge sample at full speed (14,000 rpm) for 1 minute.
- Pipet 15 µL Proteinase K into new 1.5 mL microcentrifuge tube, add 200 µL supernatant and 200 µL of Buffer AL. Vortex for 15 seconds and incubate at 70˚C for 10 minutes.
- Add 200 µL of Ethanol (96-100%) to the lysate and mix by vortexing.
- Carefully apply 600 µL lysate from last step to the QIAamp spin column. Close the cap and discard the tube containing the filtrate.

- Carefully open QIAamp spin column and add 500 µL of Buffer AW1. Centrifuge sample for 1 minute. Place the QIAamp spin column in a new 2 mL collection tube and discard the collection tube containing the filtrate.
- Carefully open the QIAamp spin column and add 500 mL of Buffer AW2. Centrifuge for 3 minute. Discard the collection tube containing the filtrate.
- Place the QIAamp spin column in a new, labelled 1.5 mL tube and pipet 100 µL of Buffer ATE directly onto the QIAamp membrane. Incubate for 1 minute at room temperature, then centrifuge for 1 minute to elute DNA. Store DNA sample at -20˚C.

Extraction of bacterial DNA from faecal samples using MO BIO Power fecal DNA isolation kit (#12830-50) according to the manufacturer's protocol:

- In a 2 mL Dry Bead Tube add 250 mg fresh faeces, or 250 µl OMNIgene.GUT faeces sample and mix with 750 µL of Bead Solution, gently vortex to mix.
- Heat Solution C1 to 60˚C until dissolved, and then add 60 µL of Solution C1 into Dry Bead Tube and vortex briefly. Heat sample at 65˚C for 10 minutes.
- Homogenize with Minilys homogenizer (Bertin Corp), speed 3 for 40 seconds.
- After homogenization, centrifuge tubes at 13,000 x g for 1 minute and transfer the supernatant to a clean 2 mL Collection tube (approximately 600-700 µL of sample).
- Add 250 µL of Solution C2 and vortex briefly to mix, incubate at 4˚C for 5 minutes.
- Centrifuge the tubes at 13,000 x g for 1 minute.
- Avoiding the pellet, transfer up to 600 µL of supernatant to a clean 2 mL Collection tube.
- Add 200 µL of Solution C3 and vortex briefly. Incubate at 4˚C for 5 minutes.
- Avoiding the pellet, transfer up to 600 µL of supernatant to a clean 2 mL Collection tube (do not transfer more than 750 µL of sample).
- Add 1.2 mL of Solution C4 to the supernatant and vortex for 5 seconds.
- Load 650 µL of supernatant onto a Spin filter and centrifuge at 13,000 x g for 1 minute. Discard the flow through and repeat until all the supernatant has been loaded onto the Spin filter.
- Add 500 µL of Solution C5 and centrifuge for 1 minute at 13,000 x g. Discard the flow through.
- Centrifuge again for 1 minute at 13,000 x g.
- Carefully place Spin filter in a clean 2 mL Collection tube.
- Add 100 µL of Solution C6 to the centre of the white filter membrane.
- Centrifuge at 13,000 x g for 1 minute to elute DNA and discard the Spin filter basket. Store DNA sample at -20˚C.
